# Effect of COVID-19 on frequency and severity of eating disorder admissions in a specialized pediatric inpatient unit

**DOI:** 10.1186/s40337-025-01385-w

**Published:** 2025-08-29

**Authors:** Shamilka Seneviratne, Michelle Polich, Maya M. Kumar, Tamara Maginot, Kyung E. Rhee

**Affiliations:** 1 University of California San Diego School of Medicine, 9500 Gilman Drive, San Diego, CA 92093 USA; 2 Department of Pediatrics, University of California San Diego School of Medicine, 9500 Gilman Drive, MC0874, San Diego, CA 92093 USA; 3 Department of Psychiatry, University of California San Diego School of Medicine, 9500 Gilman Drive, San Diego, CA 92093 USA

**Keywords:** Eating disorders, Hospitalization, Health care access, COVID-19

## Abstract

**Background:**

During the COVID-19 pandemic, youth experienced disruptions in their social development due to social distancing mandates. Research evaluating effects of isolation on adolescent mental health and eating disorders demonstrated increased ED visits during COVID. The goal of this study was to examine the effect of the COVID-19 pandemic on the number of eating disorder admissions at a large pediatric inpatient eating disorder unit and on illness severity.

**Methods:**

We conducted a retrospective chart review of patients admitted to a pediatric inpatient eating disorder unit from January 2019 through December 2021. Differences in number of admissions and patient characteristics were compared before and after the start of the COVID-19 stay-at-home mandate (i.e., “outbreak”). Interrupted time series analysis compared monthly admission trends pre- and post-outbreak. Logistic and linear regression models were created to evaluate differences in severity.

**Results:**

There were 143 patients admitted pre-outbreak and 314 patients admitted post-outbreak (84% female, 26.3% publicly insured, median age 15 years). There was a significant increase in the mean number of admissions per month post-outbreak (10.21, [SD 3.36] vs. 14.27 [SD 3.31], *p* < 0.01). Trends in admissions per month were stable prior to the COVID-19 outbreak (β_1_ = -0.38, *p* = 0.07) and increased after the start of the outbreak (β_3_ = 0.63, *p* < 0.01). Patients pre-outbreak had lower mean systolic blood pressures than post-outbreak (β = -1.90 [SE 0.82], *p* = 0.02). The odds of hypophosphatemia were lower post-outbreak (OR 0.42 [95% CI 0.21–0.81]).

**Conclusions:**

Clinical characteristics on admission were less severe post-outbreak. Closures of outpatient treatment programs may have led to lower acuity patients being hospitalized. Preserving access to robust outpatient eating disorder treatment may prevent this phenomenon during future public health crises.

## Abstract (plain language summary)

Reported cases of mental health illnesses increased during COVID-19. In this study, we aimed to analyze the effect of COVID-19 on a large, urban eating disorder unit. This study examined change in eating disorder admissions, as well as severity of symptoms, before and after the COVID-19 outbreak. While frequency of eating disorder admissions increased during the COVID-19 outbreak, this study found that severity of some symptoms decreased. Understanding the needs of this population during times of limited access to care can inform more effective, personalized care strategies that reduce long-term impacts on mental and physical health.

## Background

During adolescence, social interaction with peers is essential for normal psychosocial development and functioning [1]. Lockdown restrictions imposed during the COVID-19 pandemic disrupted school and home routines for adolescents globally, isolating them when they were developmentally vulnerable and adversely impacting their mental health [2]. Many studies have shown an increase in the prevalence of eating disorders (ED) since the onset of the pandemic, including an increase in the number of eating disorder patients requiring inpatient medical management [3–5]. However, few studies have explored the impact of the pandemic on the severity of ED symptoms at the time of admission.

Most of the studies to date regarding ED severity have been conducted in Europe or Canada. One study from Ireland found no changes in clinical severity among adolescent patients following the start of the pandemic [6]. However, several other studies have noted that adolescents were more likely to present to the hospital at a lower percentage of their treatment goal weight (TGW) and with a lower average heart rate compared to patients admitted prior to the pandemic [7–10]. Additionally, some studies have demonstrated more severe psychological symptoms (i.e., ED thoughts and behaviors) following the onset of the pandemic [11–13]; one study found that up to 81% of adolescents already in treatment for eating disorders reported worsening of ED-related thoughts and behaviors during the pandemic [11]. However, no studies have examined a broad spectrum of physiologic *and* psychosocial markers of severity (e.g., lab results, cardiac function, and evidence of psychological and social distress) among hospitalized youth with EDs.

The goal of this study was to examine the potential influence of the COVID-19 pandemic on (1) the average monthly number of admissions and (2) physiologic and psychosocial markers of ED severity, among pediatric and young adult patients admitted to a specialized inpatient eating disorder unit before and after the start of the pandemic.

## Methods

### Study population

We conducted a retrospective chart review of patients with Anorexia Nervosa (AN), restricting or binge-purge subtype, Atypical AN, or Avoidant Restrictive Food Intake Disorder (ARFID), admitted to the Medical Behavioral Unit (MBU) at Rady Children’s Hospital San Diego from January 2019 through December 2021. The MBU is a specialized eating disorder unit that cares for children, adolescents, and young adults with eating disorders and medical complications of malnutrition. For admission, patients had to meet at least one of the following criteria: weight less than 75% of treatment goal weight (TGW), resting heart rate < 50 bpm while awake, hypotension (blood pressure < 90/50), orthostatic hypotension (drop in systolic blood pressure ≥ 20 mmHg or drop in diastolic blood pressure ≥ 10 mmHg with standing), orthostatic tachycardia (increase in heart rate by ≥ 20 bpm with standing), syncope, severe dehydration (clinical and/or laboratory markers of dehydration in combination with inability to drink adequate fluids), rapid weight loss of ≥ 3 pounds per week, and/or abnormal laboratory or electrocardiographic findings associated with malnutrition. TGW was calculated using past growth and development trajectory or using median BMI if past history was unknown. If a patient had multiple admissions during the study period, only the initial encounter was used. Patients were divided into 2 groups based on their date of admission: pre-outbreak (January 1, 2019 - March 31, 2020) and post-outbreak (April 1, 2020 – December 1, 2021). These dates were chosen because a state of emergency was declared in California on March 4, 2020 and a statewide stay-at-home mandate was issued on March 19, 2020; therefore, April 2020 was the first full month after lockdown measures were implemented in California [14]. At this time, all outpatient eating disorder programs were expected to be closed. This study was approved by the UC San Diego Human Research Protections Program.

### Measures

Data was abstracted from the electronic medical records (EMRs) of all patients admitted to the MBU within the study period. Our first primary outcome was the average monthly number of admissions over time. The second primary outcomes reflected severity of illness, which was measured by several physiologic and psychosocial indicators. Physiologic status was assessed using the following variables: percentage of TGW at admission; lowest overnight heart rate, lowest blood pressure, and greatest change in orthostatic blood pressure and heart rate observed during the first 3 days of admission; and proportion of patients with abnormal electrolyte values during the first 7 days of admission (serum potassium < 3.5 mEq/L, serum phosphorus < 3.0 mEq/L, serum magnesium < 1.8 mEq/L, serum glucose < 70 mg/dL, hemoglobin < 12.5 g/dL, aspartate aminotransferase [AST] > 36 U/L, alanine aminotransferase [ALT] > 35 U/L).

The need for nasogastric/nasojejunal feeding tubes or physical restraints at any time during admission was used as an indirect marker of psychological severity because these tools were only used when patients were struggling to eat or had severe psychological distress related to their eating disorder or other psychiatric comorbidities. Involvement of child welfare services during admission was used as an indirect measure of social or family distress. Rate of readmission within 90 days of discharge was also evaluated.

Demographic information obtained from the EMR included gender, age at the time of admission, ethnicity, and insurance status. Ethnicity was categorized as non-Hispanic White, Hispanic, or Other non-Hispanic/non-White. Insurance status was categorized as commercial insurance or government insurance (Medicaid, Medicare).

### Statistical analysis

Demographic information and clinical characteristics of all participants were described using frequencies and means with standard deviations where appropriate. To examine differences in severity from before and after the outbreak, bivariate analyses were performed using chi-square for categorical variables and independent sample *t* tests for continuous variables. Separate multivariable logistic and linear regression models were created for each outcome variable that had a p-value < 0.05 in the bivariate analyses. All models were adjusted for age, gender, insurance status, and if the patient had any prior MBU admissions before the study period.

To examine if there were differences in the average monthly number of admissions before and after the outbreak, an interrupted time series analysis was used. The model is represented by: Y = B_0_ + B_1_*T + B_2_*D + B_3_*P + e. The outcome variable (Y) represents frequency of admissions, defined as the number of admissions to the MBU per month. Time (T) was defined as the number of months from the start of the study, ranging from 1 to 36 months. B_1_ represents the trend in admission rates prior to the COVID pandemic. The two groups were represented by a dummy variable with 0 and 1 categories for months before and months after the COVID-19 outbreak, respectively (D). B_2_ therefore represents level change between pre-outbreak and post-outbreak months. The time passed after the start of the COVID-19 outbreak (P) is represented by a continuous variable, assigned 0 before the COVID-19 outbreak and increasing by the number of months after the COVID-19 outbreak and lockdown measures began in March 2020. B_3_ represents change in slope before and after the start of the COVID-19 outbreak. Autocorrelation was assessed using the Durbin-Watson test and corrected for using stepwise autoregression. All analyses were performed using SAS (v9.4; Cary, NC) and p-value < 0.05 was considered significant.

## Results

### Demographic information

A total of 457 patients were included over 36 months (84.2% female, 26.3% publicly insured, median age of 15 years (SD 3.55 years, range 4–32 years). (Table 1) There were 143 patients admitted during the 15-month pre-outbreak period and 314 patients admitted during the 21-month post-outbreak period. There were no significant differences in demographic characteristics including gender, age, insurance type, and race/ethnicity.

**Table 1:** Patient characteristics (*n* = 457)

	Pre-outbreak (*n* = 143)	Post-outbreak (*n* = 314)	*p*-value
Gender, n (%)			
Male	20 (13.99)	39 (12.42)	0.90
Female	119 (83.22)	266 (84.71)	
Transgender or non-binary	4 (2.80)	9 (2.87)	
Age (years), n (%)			
<15	57 (39.86)	128 (40.76)	0.86
≥15	86 (60.14)	186 (59.24)	
Insurance, n (%)			
Commercial^a^	107 (74.83)	230 (73.25)	0.72
Government	36 (25.17)	84 (26.75)	
Race/Ethnicity^b^, n (%)			
NH-White	85 (59.86)	174 (57.24)	0.76
NH-Other	20 (14.08)	51 (16.78)	
Hispanic	37 (26.06)	79 (25.99)	
First MBU Admission, n (%)	132 (92.31)	308 (98.09)	< 0.01

NOTE: Abbreviations: NH = Non-Hispanic, MBU = Medical Behavioral Unit

^a^Includes private and Military insurance

^b^Missing 11 patients with unknown race and/or ethnicity (*n* = 446)

### Frequency of admissions

**Fig. 1:**
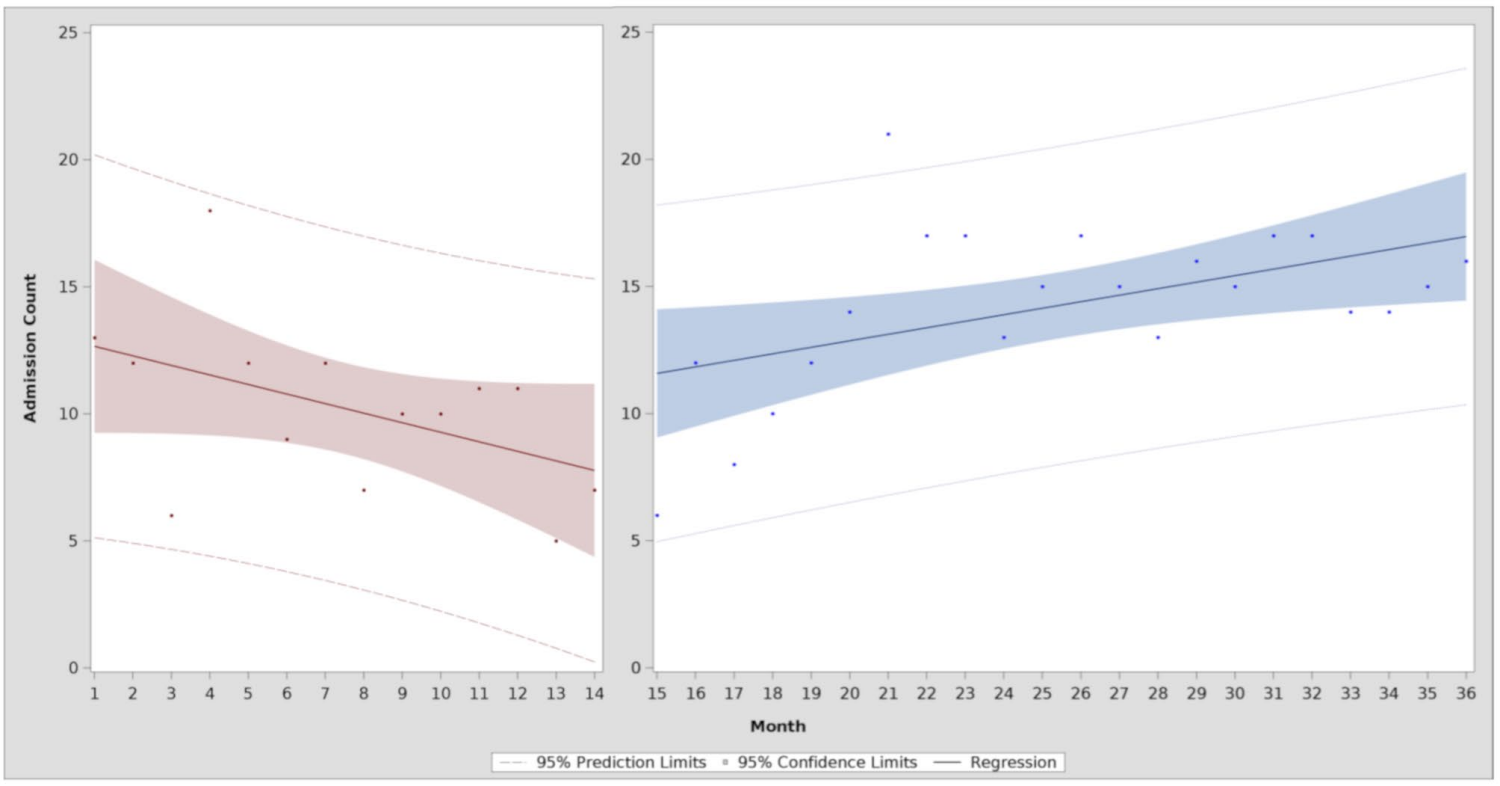
Interrupted Times Series: Admission count per month. Trends in the mean number of monthly admissions before and after the outbreak were examined using interrupted time series analysis. Trend prior to the COVID-19 outbreak was decreasing but not significant (β_1_ = -0.38, *p* = 0.07). There was no significant change from February 2020 to March 2020 (β_2_ = 3.55, *p* = 0.09). The trend in mean number of admissions per month increased after March 2020 (β_3_ = 0.63, *p* = 0.008)

The average monthly number of admissions increased from a mean of 10.21 per month (SD 3.36) pre-outbreak to 14.27 per month (SD 3.31) post-outbreak (*p* < 0.01). Trends in admissions per month were stable prior to the COVID outbreak (β_1_ = -0.38, *p* = 0.07). There was no significant change from February 2020 to March 2020 (β_2_ = 3.55, *p* = 0.09). The average number of admissions per month increased after the implementation of lockdown measures in March 2020 (β_3_ = 0.63, *p* < 0.01). (Fig. 1)

### Eating disorder severity

The percent of TGW at the time of admission was similar before and after the outbreak. Bivariate analysis showed that the mean lowest systolic blood pressure during the first 3 days of admission was significantly lower pre-outbreak than post-outbreak (88.22 [SD 8.97] vs. 90.16 [SD 7.60], *p* = 0.03). (Table 2) Prevalence of hypophosphatemia was significantly higher pre-outbreak than post-outbreak (14.7% vs. 6.4%, *p* < 0.01). The percentage of patients presenting with their first admission to the eating disorder unit was also significantly lower pre-outbreak compared to post-outbreak (92% vs. 98%, *p* < 0.01). No other physiologic differences were noted between time periods. There were also no differences in the use of nasogastric/nasojejunal feeding tubes, application of physical restraints, or child welfare service involvement.

**Table 2:** Difference in measures for patients hospitalized Pre- and Post- COVID-19 outbreak (*n* = 457)

	Pre-outbreak (*n* = 143)	Post-outbreak (*n* = 314)	*p*-value
	Mean (SD)	Mean (SD)	
Admission %TGW^a^	0.78 (11.15)	0.80 (11.76)	0.23
Vital Signs			
Lowest overnight HR	46.08 (10.24)	46.04 (11.05)	0.97
Lowest Systolic BP	88.22 (8.97)	90.16 (7.60)	0.03
Lowest Diastolic BP	45.59 (6.76)	46.43 (6.52)	0.21
	N (%)	N (%)	
Orthostatic Vital Signs^b^			
Drop in systolic BP ≥ 20 mmHg	13 (9.09)	35 (11.15)	0.51
Drop in diastolic BP ≥10 mmHg	19 (13.29)	42 (13.38)	0.98
Increase in heart rate by ≥ 20 bpm	124 (86.71)	272 (86.62)	0.98
Laboratory Results			
Hypokalemia (K < 3.5 mg/dL)	26 (18.18)	39 (12.42)	0.10
Hypophosphatemia (Phos < 3 mg/dL)	21 (14.69)	20 (6.37)	0.004
Hypomagnesemia (Mg < 1.8 mg/dL)	29 (20.28)	53 (16.88)	0.38
Hypoglycemia (glucose < 70 mg/dL)	39 (27.27)	89 (28.34)	0.81
Neutropenia	13 (9.09)	35 (11.15)	0.51
Anemia (Hgb < 12.5 g/dL)	46 (32.17)	105 (33.44)	0.79
Elevated AST/ALT ratio	69 (48.25)	158 (50.32)	0.68
Elevated AST (AST > 36 U/L)	65 (45.45)	156 (49.68)	0.40
Elevated ALT (ALT > 35 U/L)	30 (20.98)	44 (14.01)	0.06
NG/NJ	44 (30.77)	84 (26.75)	0.38
CPS involvement	16 (11.19)	34 (10.83)	0.91
Restraint Orders	7 (4.90)	14 (4.46)	0.84
90-day readmission	5 (3.50)	17 (5.41)	0.37

NOTE: Abbreviations: TGW = treatment goal weight, BP = blood pressure, HR = heart rate, BP = blood pressure, AST = aspartate transaminase, ALT = alanine transaminase, NG = nasogastric tube, NJ = nasojejunal tube, CPS = child protective services

^a^Missing 6 patients without treatment goal weight documented (*n* = 451)

^b^Orthostatic hypotension (drop in systolic blood pressure or drop in diastolic blood pressure), Orthostatic tachycardia (increase in heart rate with standing))

In the multivariable analyses, pre-outbreak patients continued to have a significantly lower mean SBP than post-outbreak patients (β = -1.90 [SE 0.82], *p* = 0.02). (Table 3) The odds of hypophosphatemia remained significantly lower post-outbreak (OR 0.42 [95% CI 0.21–0.81]).

**Table 3:** Multivariable analysis of physiologic indicators of eating disorder severity

	Pre-outbreak	Post-outbreak	*p*-value
Lowest SBP, adj mean (95% CL)	88.22 (88.08–88.37)	90.16 (90.06–90.26)	0.02
Low Phosphorous, OR [95% CL]	Ref	0.42 [0.21–0.81]	0.01

NOTE: Abbreviations: SBP = systolic blood pressure

Separate multivariable models were conducted for each outcome of interest, adjusted for age, gender, insurance, and first MBU admission or not

## Discussion

This study adds to the existing literature by assessing both changes in admission frequency and a wider spectrum of characteristics related to severity, including physiologic and psychosocial markers. Previous studies examining patients hospitalized with an eating disorder have typically found that more youth were hospitalized following the COVID-19 outbreak, with many of them presenting with lower weights and more cardiovascular compromise than patients admitted before the COVID-19 outbreak [8, 15]. In this study, we similarly found that the average number of patients admitted per month was significantly higher after the outbreak. However, their severity of illness did not appear to worsen; in fact, some markers of severity were better among patients hospitalized post-outbreak. In this analysis, controlling for demographic characteristics and whether this was their first admission or not, patients admitted after the outbreak had a higher systolic blood pressure and lower odds of hypophosphatemia than patients admitted pre-outbreak.

There are several possible reasons for this finding. First, patients may have had more difficulty accessing outpatient ED treatment following the COVID-19 outbreak. Many programs, including outpatient mental health and dietitian appointments, partial hospitalization programs, and residential treatment programs suspended their in-person services to reduce viral transmission and comply with lockdown mandates. A study among a large collaborative of 15 U.S. eating disorder programs showed that there was almost a 40% decrease in the number of outpatient assessments conducted among sites during April 2020, the first month following implementation of lockdown measures in most states [8]. Reduced access to outpatient care may have resulted in greater reliance on emergency rooms and inpatient services for care. This may also explain the greater number of patients who had their first admission during the outbreak.

Second, while most outpatient programs eventually created virtual options for treatment during the pandemic, patients, families, and clinicians have expressed challenges with virtual eating disorder treatment, despite recognition that it was superior to forgoing treatment during lockdown [10]. Compared to other psychiatric conditions, the treatment of EDs is significantly more difficult to convert to virtual treatment platforms given the greater burden placed on patients and caregivers to choose and provide adequate meals, implement supervision around eating, and intervene when ED behaviors occur. Furthermore, patients are frequently unable to obtain measurements of weight and vital signs for clinicians to monitor [15–17]. In some cases, the use of video conferencing for health care may have even heightened body awareness among patients with EDs [18–19]. As such, even if outpatient treatment options were available, patients may have struggled to engage with them, which could have led to a greater need for hospital admissions.

Lastly, even if outpatient treatment programs remained operational, a large increase in wait times was seen across programs due to both increased demand for and decreased supply of outpatient services [20–21]. For example, one study showed that the average wait time to see an eating disorder treatment therapist increased by three to five months during the pandemic [22]. Thus, timely recognition and outpatient treatment was more challenging to attain during the pandemic, which may have led to earlier presentation to inpatient facilities for treatment and stabilization.

While our study evaluated the frequency and severity of admissions from a large catchment region in Southern California, there were limitations to the study. First, our findings were limited by data from a single site and the available data that could be extracted from the EMR. Data was collected from a specialized inpatient ED unit, which may limit generalization to hospitals without specialized ED programs. While we were able to examine several indirect indicators of psychosocial distress, we did not have data available from standardized psychological assessments to better characterize the degree of cognitive or emotional symptom severity before and after the outbreak. Additionally, while statistically significant, the differences in blood pressure were small, about 2 mm Hg, which may not necessarily be clinically significant.

## Conclusion

This study assessed changes to a broad spectrum of both physiologic and psychosocial markers of severity among youth hospitalized with EDs following the COVID-19 outbreak. Similar to previous studies, we demonstrated an increase in the monthly number of ED hospital admissions post-outbreak. However, we also demonstrated a *decrease* in the severity of physiologic indicators during the post-outbreak period. This finding may have been related to an increased reliance on inpatient services due to decreased availability and accessibility of outpatient treatment options during the lockdown. These results highlight the importance of protecting access to both inpatient and outpatient ED services even in the midst of a public health crisis. Future studies are needed to assess the longer-term impacts of the pandemic experience on the prevalence and severity of eating disorders among children and adolescents.

## Data Availability

Data Sharing: Deidentified individual-level participant data and code book will be made available after publication to researchers who provide a methodologically sound proposal. Proposals should be submitted to Dr. Kay Rhee at k1rhee@health.ucsd.edu for review and approval. Data use agreements will be required.

## Abbreviations

ED: Eating disorder
TGW: Treatment goal weight
MBU: Medical behavioral unit
EMR: Electronic medical record
AST: Aspartate aminotransferase
ALT: Alanine aminotransferase
